# Insight into the Ebola virus nucleocapsid assembly mechanism: crystal structure of Ebola virus nucleoprotein core domain at 1.8 Å resolution

**DOI:** 10.1007/s13238-015-0163-3

**Published:** 2015-04-25

**Authors:** Shishang Dong, Peng Yang, Guobang Li, Baocheng Liu, Wenming Wang, Xiang Liu, Boran Xia, Cheng Yang, Zhiyong Lou, Yu Guo, Zihe Rao

**Affiliations:** State Key Laboratory of Medicinal Chemical Biology and College of Pharmacy, Nankai University, Tianjin, 300071 China; College of Life Sciences, Nankai University, Tianjin, 300071 China; Tianjin International Joint Academy of Biotechnology & Medicine, Tianjin, 300457 China

**Keywords:** *Filoviridae*, Ebola virus, nucleoprotein, nucleocapsid, crystal structure, assembly mechanism

## Abstract

Ebola virus (EBOV) is a key member of *Filoviridae* family and causes severe human infectious diseases with high morbidity and mortality. As a typical negative-sense single-stranded RNA (−ssRNA) viruses, EBOV possess a nucleocapsid protein (NP) to facilitate genomic RNA encapsidation to form viral ribonucleoprotein complex (RNP) together with genome RNA and polymerase, which plays the most essential role in virus proliferation cycle. However, the mechanism of EBOV RNP formation remains unclear. In this work, we solved the high resolution structure of core domain of EBOV NP. The polypeptide of EBOV NP core domain (NP_core_) possesses an N-lobe and C-lobe to clamp a RNA binding groove, presenting similarities with the structures of the other reported viral NPs encoded by the members from *Mononegavirales* order. Most strikingly, a hydrophobic pocket at the surface of the C-lobe is occupied by an α-helix of EBOV NP_core_ itself, which is highly conserved among *filoviridae* family. Combined with other biochemical and biophysical evidences, our results provides great potential for understanding the mechanism underlying EBOV RNP formation via the mobility of EBOV NP element and enables the development of antiviral therapies targeting EBOV RNP formation.

## INTRODUCTION

The members of filovirus family, including Ebola virus (EBOV) and Marburg virus (MARV) and Lloviu virus (LLOV) (Kuhn et al., 2010), cause highly lethal hemorrhagic fever in human beings with extremely high morbidity and mortality. EBOV is typically found in Central Africa, but re-emerged in Western Africa in 2014 to cause a worldwide-spreading outbreak spreading. Till April 2015, 25,591 suspected cases and 10,602 dead cases were reported (http://www.cdc.gov/vhf/ebola/outbreaks/2014-west-africa/case-counts.html). Although several chemical agents, antibodies and vaccines are found to inhibit EBOV in animal or human, there is no therapeutics with high efficacy can be provided for clinical usage.

EBOV is a typical non-segmented negative-sense single-stranded RNA (−ssRNA) virus (Muhlberger et al., 1999). The single-stranded RNA genome of EBOV encodes the surface glycoprotein (GP), an RNA-dependent RNA polymerase (RdRp), a nucleocapsid protein (NP), as well as viral protein (VP)35, VP40, VP30 and VP24 (Muhlberger et al., 1999). Similar to other −ssRNA viruses, the RNA genome of EBOV cannot exist as a naked form, but must be encapsidated by NP and further form a ribonucleoprotein (RNP) complex together with RdRp (Sun et al., 2012; Zhou et al., 2013). After entry into the cytoplasm through membrane fusion mediated by glycoproteins, the RNP is released from the virion and serves as the template with which the copackaged RdRp transcribes mRNAs from the viral genome in the RNP. In the later stage of virus replication, complementary positive-strand RNA (cRNA) is produced in the form of an RNP. The RNP serves as the template for replication that generates viral genomic RNA in the form of an RNP ready to be packaged in the virion. Throughout the entire virus replication cycle of a −ssRNA virus, the genome length viral RNA (cRNA or viral genomic RNA) is only present in the form of an RNP that either serves as a template for RNA synthesis or is packaged in the virion. Therefore, the formation and correct function of RNP is essential for −ssRNA virus proliferation (Sun et al., 2012; Zhou et al., 2013; Ruigrok et al., 2011).

Structural knowledge of −ssRNA virus RNPs was initiated by studying non-segmented −ssRNA viruses, including Borna disease virus (BDV) (Rudolph et al., 2003), rabies virus (Albertini et al., 2006), vesicular stomatitis virus (VSV) (Green et al., 2006), and respiratory syncytial virus (RSV) (Tawar et al., 2009), and this knowledge has been greatly enhanced by recent investigations of segmented −ssRNA viruses, including arenaviruses (Hastie et al., 2011a, b; Qi et al., 2011), bunyaviruses (Dong et al., 2013; Ferron et al., 2011; Guo et al., 2012; Jiao et al., 2013; Li et al., 2013; Niu et al., 2013; Raymond et al., 2010), and influenza virus (Ng et al., 2008; Ye et al., 2006; Arranz et al., 2013; Chenavas et al., 2013; Moeller et al., 2013). Furthermore, advances in the visualization of native or authentic RNP through electron microscopy led to the understanding of the dynamic processes of RNP formation at molecular level. In particular, recent results have clearly demonstrated that viral NP could be directly used as the target for antiviral development (Gerritz et al., 2011; Kao et al., 2010), raising a great potential to find new antiviral agents with novel mechanism against drug resistance occurred in traditional antiviral drugs. Recent work on cryo-electron microscopy and tomography help us visualize *Filoviridae* virus particles and formation of nucleocapsid (Bharat et al., 2012; Bharat et al., 2011). However, the structural details of EBOV NP and the molecular mechanism of EBOV RNP formation are largely unknown. The exclusive structural information is that a recent work identified the boundaries of N- and C-terminal domains of EBOV NP and solve the crystal structure of the C-terminal domain spanning residues 641–739 (Dziubanska et al., 2014). However, the structure of C-terminal domain of EBOV NP did not hint to the biological function of this region. All of these promote us to initiate the biochemical and structural analysis on EBOV NP to achieve the understanding of the mechanism of EBOV RNP formation and explore the potential drug target for the discovery of anti-EBOV agents.

## RESULTS

### Biochemical analysis of the recombinant core domain of EBOV NP

Previous studies have demonstrated that N-terminal truncation of EBOV NP resulted the loss-of-function in NP-NP interaction and the first 450 amino-acid of EBOV NP, forming the core domain, is sufficient for the formation of nucleocapsid-like structures and viral genome replication (Watanabe et al., 2006). However, most of previous expression and purification work aimed for structural investigation failed because of the tendency to oligomerization and precipitate. To screen the best construct suitable for the crystallographic work on the core domain of EBOV NP, we generate a series of truncation and mutation constructs. All constructs containing the first 30 amino acids of EBOV NP result to high tendency to oligomerize and precipitate, even in ultra-high ion strength condition.

The best construct with the highest expression yield and biochemical features covers the region of residue 36–351 of EBOV NP (named NP_core_ hereafter). Moreover, this region is highly conserved not only in different strains of EBOV, but also in Marburg virus and Lloviu virus, another two representative member in *Filoviridae* family. In particular, this region was revealed to exert the both the function of RNA binding and further NP oligomerization to form high-order RNP. We therefore performed further crystallographic and biochemical investigation on this NP_core_ region.

**Figure 1:**
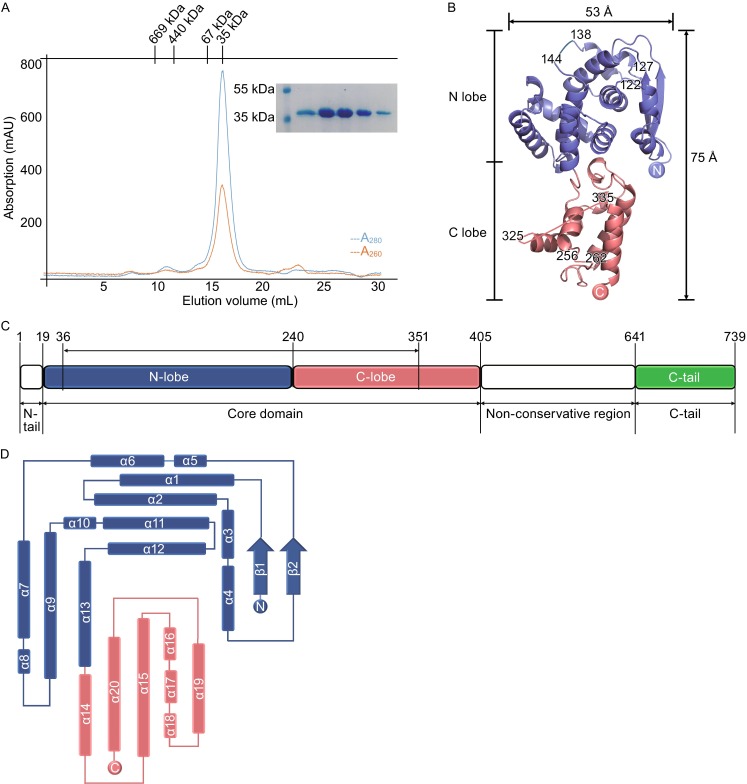
**Purification and structure of EBOV NP**_**core**_. (A) Size exclusion chromatography (SEC) of EBOV NP_core_. The sample containing EBOV NP_core_ was injected into a Superdex-200 column. The molecular weights of standard protein markers are shown on the top. Blue and red lines denote A_280_ and A_260_, respectively. SDS-PAGE analysis of the peak fractions is shown inset. (B) Overall structure of EBOV NP_core_. The polypeptide of EBOV NP_core_ is shown as colored cartoon. The N-lobe and C-lobe are colored as blue and red, respectively. Missing residues are linked by dotted lines. (C) Schematic diagram of the domain organization in the primary sequence of EBOV NP. N-lobe, C-lobe, and C-tail are colored as slate blue, salmon red, and green, respectively. N-tail and non-conservative region are colored as blank. (D) Topology diagram of EBOV NP_core_ helices are presented as rectangulars and strands are shown as arrows. The color scheme is the same as that in (B) and (C)

Because binding RNA and oligomerization are two key features for virally encoded NP, we further analyzed the solution property of EOBV NP_core_. The EBOV NP_core_ was expressed in *Escherichia coli* and purified under physiological conditions. The retention volume of the recombinant EBOV NP_core_ protein in size-exclusion chromatography peaks at 16 mL (Fig. 1A), corresponding to a molecular weight to 35 kDa. SDS-PAGE analysis indicated that the major peaks contained a protein with expected size for EBOV NP_core_, while the A_280_/A_260_ UV absorption ratio of 1.6 demonstrated that there was no nucleic acid binding to EBOV NP_core_ (Fig. 1A). These results revealed that the recombinant EBOV NP_core_ exists as a monomeric state in solution and do not contain nucleic acids acquired from expression host cells.

### Overall structure of EBOV NP_core_

The EBOV NP_core_ was successfully crystallized, and the crystal structure was subsequently determined using the single-wavelength anomalous dispersion (SAD) method and refined to a high resolution of 1.8 Å with a final *R*_work_ of 19.2% (*R*_free_ = 22.3%) (Table 1).

**Table 1:** **Data collection and refinement statistics**

Parameters	Native	SeMet
*Data collection statistics*		
Cell parameters		
* a* (Å)	59.8	59.3
*b* (Å)	162.9	162.9
*c* (Å)	31.3	31.1
*α*, *β*, *γ* (°)	90.0, 90.0, 90.0	90.0, 90.0, 90.0
Space group	*P2*_*1*_ *2*_*1*_ *2*	*P2*_*1*_ *2*_*1*_ *2*
Wavelength used (Å)	0.9785	0.9785
Resolution (Å)	40.7–1.79 (1.86–1.79)^c^	40.1–2.4 (2.49–2.40)
No. of all reflections	312,740 (14,770)	158,723 (7888)
No. of unique reflections	29,588 (1477)	12,497 (580)
Completeness (%)	99.16 (93.53)	99.50 (96.14)
Average *I*/*σ* (*I*)	20.04 (3.94)	34.08 (8.9)
$R_{\text{merge}}^{\text{a}}$ (%)	8.4 (54.6)	16.6 (45.1)
Refinement statistics		
No. of reflections used (*σ*(*F*) > 0)	29492	
$R_{\text{work}}^{\text{b}}$ (%)	19.19	
$R_{\text{free}}^{\text{b}}$ (%)	22.30	
r.m.s.d. bond distance (Å)	0.011	
r.m.s.d. bond angle (º)	1.19	
Average B-value (Å^2^)	39.5	
No. of protein atoms	2512	
No. of ligand atoms	0	
No. of solvent atoms	293	
Ramachandran plot		
Res. in favored regions (%)	98.23	
Res. in generously allowed regions (%)	1.77	
Res. in disallowed regions (%)	0	

^a^ *R*_*merge*_ = Σ_h_Σ_l_ | *I*_ih_ − <*I*_h_> |/Σ_h_Σ_I_ <*I*_h_>, where <*I*_h_> is the mean of the observations *I*_ih_ of reflection h

^b^ *R*_*work*_ = Σ(||*F*_p_(obs)| − |*F*_p_(calc)||)/Σ|*F*_p_(obs)|; *R*_*free*_ is an *R* factor for a pre-selected subset (5%) of reflections that was not included in refinement

^c^Numbers in parentheses are corresponding values for the highest resolution shell

In the structure of EBOV NP_core_, one molecule is observed in the crystallographic asymmetric unit. Further analysis suggests that the intramolecular interactions are associated with crystal packing and the monomer should be the biological unit. All residues of EBOV NP_core_ polypeptide were be built into the final model, except three short gaps (residues A123–S126, E139–T143, K257–R263), and one loop region covering A326–V334 cannot be traced due to the lack of interpretable electron density, indicating their intrinsic structural flexibility (Fig. 1B).

The structure of EBOV NP_core_ presents a novel protein folding within viral NP family and displays a compact architecture with dimensions approximately of 53 Å × 40 Å × 75 Å. EBOV NP_core_ is featured by two relatively separate portions, an N-lobe (V36–R240), and a C-lobe (F241–E351) (Fig. 1B and 1C). Both two domains are predominantly composed of α-helices, in which the N-lobe consisted of thirteen α-helices and two β-strands and the C-lobe consisted of seven α-helices (Fig. 1B and 1D). Similar with most virus-encoded NPs, the N- and C-lobes clamp a highly positively charged groove to encapsidate viral RNA.

### The RNA binding groove of EBOV NP_core_

The A_280_/A_260_ ratio of EBOV NP_core_ is approximate 1.6 and indicates no RNA binding occurred during the expression and purification of EBOV NP_core_ (Fig. 1A). In consistency, no continuous nucleic acids electron density could be observed on the surface of EBOV NP_core_.

**Figure 2:**
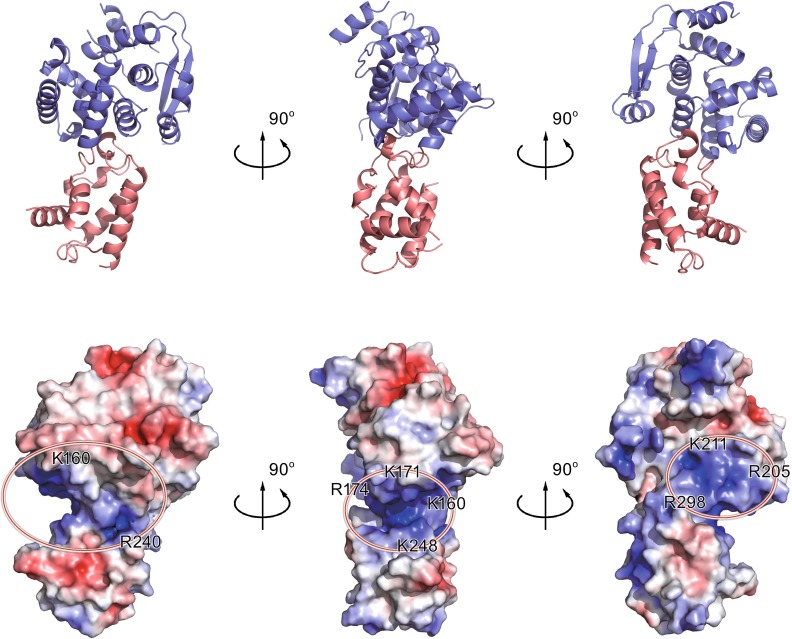
**Potential RNA binding region of EBOV NP**_**core**_. (A) Cartoon representation of EBOV NP_core_. The N-lobe and C-lobe are colored as slate blue and salmon red, respectively. (B) Electrostatic surface potential of EBOV NP_core_. The electrostatic surface potential of EBOV NP_core_ was calculated using APBS tools, with limits ±5 kbT/ec. Positive residues are highlighted by red circle on EBOV NP_core_, suggesting the presence of several positively charged grooves that may be involved in RNA binding

However, highly positively charged pockets on the molecular surface of EBOV NP_core_ coordinated the investigation on the RNA binding site (Fig. 2). There are two positive-charged regions located in the structure of EBOV NP_core_, a major one and a minor one. The major positive-charged crevice is located in the interface of N-lobe and C-lobe, in which K160, K171, R174, and K248 contribute the most essential positive charge. An additional minor positive-charged region is adjacent to the major crevice and is consisted by R205, K211, and R298. Positive-charged residues, including lysine, arginine, and histidine residues, are known to interact with the ribose and phosphate moieties of nucleic acid and thus dominate the RNA encapsidation process of viral NPs (Albertini et al., 2006; Green et al., 2006; Hastie et al., 2011; Guo et al., 2012; Li et al., 2013; Ariza et al., 2013; Raymond et al., 2012; Reguera et al., 2013). To be consistent with this structural observation in the major groove in EBOV NP_core_, previous works have demonstrated that deletion of these basic residues or substitutions to alanine residues significantly impaired the RNA binding affinity of EBOV NP or EBOV replication (Muhlberger et al., 1999; Watanabe et al., 2006; Noda et al., 2010; Huang et al., 2002; Leung et al., 2015). Taken together, these structural and biochemical evidences designated that the RNA binding groove of EBOV NP is located in the interface of N- and C-lobes and demonstrated the essential role of the key basic residues for RNA binding and EBOV proliferation.

### Structural comparison of EBOV NP_core_ with other NPs in *Mononegavirales*

**Figure 3:**
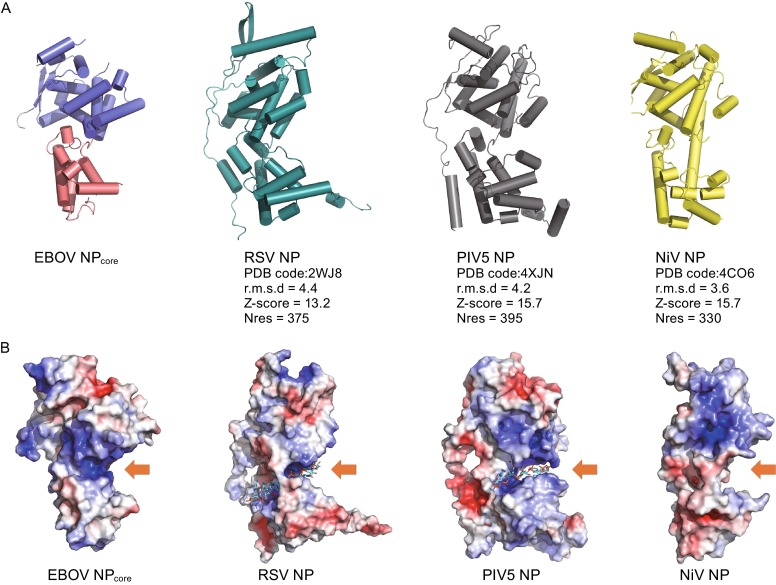
**Structural comparison of NPs from** ***Mononegavirales*** **viruses**. (A) Structural comparison of EBOV NP_core_ with RSV, PIV5, and NiV NP. NPs are displayed as cartoon. RSV (PDB code: 2WJ8), PIV5 (PDB code: 4XJN), and NiV (PDB code: 4CO6) NPs are colored cyan, gray, and yellow, respectively. Alignment information is listed under each molecule. All molecules are aligned to the structure of EBOV NP_core_ and shown in the same orientation. (B) Electrostatic potential comparison of EBOV NP_core_ with RSV, PIV5, and NiV NP. Positively charged pockets for RNA binding are indicated with orange arrows. RNA molecules in RSV and PIV5 NPs are shown as colored sticks

DALI (Holm and Rosenstrom, 2010) and SSM (Krissinel and Henrick, 2004) analysis revealed that EBOV NP_core_ presents structural similarity with viral NPs encoded by the members of *Mononegavirales* order, including RSV (Tawar et al., 2009), parainfluenza virus 5 (PIV-5) (PDB code: 4XJN), and Nipah virus (NiV) (Yabukarski et al., 2014), and etc. Alignment of EBOV NP_core_ with RSV NP structures gives an overall root-mean-square deviation (r.m.s.d.) of 4.4 Å for all Cα atoms of the 375 aligned residues, while the alignment with PIV5 NP gives an r.m.s.d. of 4.2 Å for 395 residues and NiV NP gives an r.m.s.d. of 3.6 Å for 330 residues (Fig. 3A).

Not only NPs of RSV, PIV-5, and NiV, the overall structure of all *Mononegavirales* NPs shows similar topology, despite of low primary sequence homology. All of these NPs include an N-lobe and a C-lobe to clamp the RNA binding site on interface. However, the formations of RNP achieved by viral NPs are variable. The most striking difference is that the continuous nucleic acid chains are found to be bound in the inner side of NP-RNA particles of rabies virus (Albertini et al., 2006) and VSV (Green et al., 2006), but the bound RNAs are exposed to the outer side of NP-RNA particles of RSV (Tawar et al., 2009) and PIV5 NP. According to the structure homologies of EBOV NP_core_ with NPs encoded by RSV and PIV5, we speculate the RNA binding position of EBOV NP should adopt same pattern. Notably, besides the core domain, EBOV NP accommodates an non-conservative region and a C-terminal tail, which is nearly 200 amino-acid length, suggesting EBOV NP may have additional biological functions beyond RNA binding and oligomerization to form high-ordered RNP. Actually, a breakthrough in the understanding of virally encoded NP is that Lassa fever virus (LASV) (Hastie et al., 2011; Qi et al., 2011) and Crimean-Congo hemorrhagic fever (CCHFV) (Guo et al., 2012) NPs do have an enzymatic activity. These indicate that the further analysis on the extension parts of EBOV NP is necessary to dissect its precise biological roles.

### Helix-20 in EBOV NP_core_ indicates an essential hydrophobic pocket

**Figure 4:**
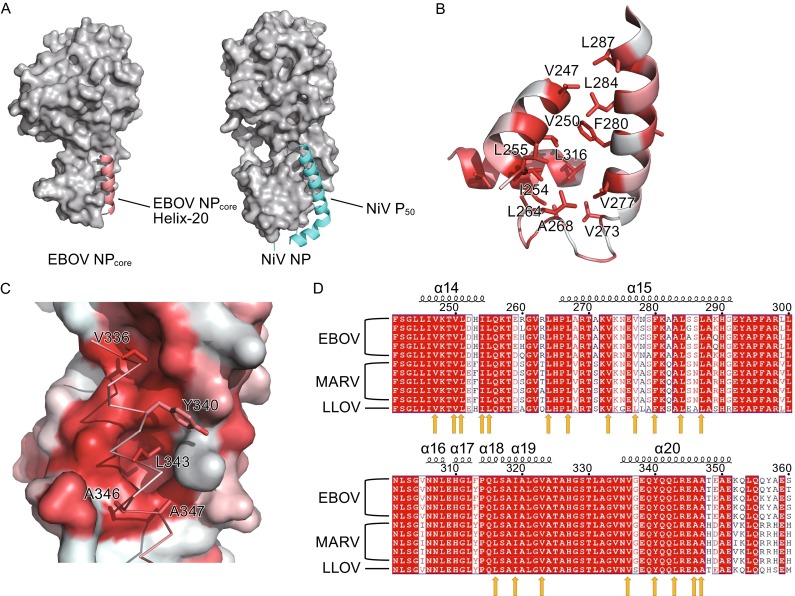
**Helix-20 in EBOV NP**_**core**_ **indicates an essential hydrophobic pocket**. (A) Comparison of EBOV NP_core_ and NiV NP. Both structures are shown as surface and colored gray. EBOV NP_core_ Helix-20 and NiV P_50_ are shown as cartoon, colored salmon red and cyan, respectively. (B) Formation of the hydrophobic pocket. Main chain is shown in cartoon and hydrophobic residues are shown in stick, colored in red by hydrophobicity. (C) Interaction of Helix-20 with hydrophobic pocket. Hydrophobic pocket are shown in surface, Helix-20 are displayed in ribbon and stick, colored in red by hydrophobicity. (D) Primary sequence alignment of members of the *filoviridae* family. Highly conserved residue in Helix-14, Helix-15, Helix-19, and Helix-20 are indicated by yellow arrow

A critical step for −ssRNA RNP function is that NP releases the encapsidated RNAs and transfers RNAs to the catalytic center of RdRp for polymerase reaction. Recent works on VSV (Green and Luo, 2009), NiV (Yabukarski et al., 2014), and arenavirus (Kranzusch and Whelan, 2011) have revealed several key co-factors or chaperones to regulate this key process. For example, a phosphoprotein (P) was shown to be the key regulator to VSV RNP formation and the C-terminal domain of P binds primarily to the C-lobe of VSV NP within NP-RNA particles (Green and Luo, 2009). Moreover, in the structure of NiV NP-P complex, a peptide derived from P was determined to locate at one hydrophobic crevice, protecting host cells against viral replication by inhibiting viral RNP formation (Yabukarski et al., 2014). Interestingly, the superimposition of EBOV NP_core_ with NiV NP-P complex suggests a similar hydrophobic pocket (Fig. 4A). This hydrophobic pocket lies on the C-lobe of EBOV NP_core_, which is adjacent to the RNA binding groove (Fig. 4A).

In EBOV NP_core_, the hydrophobic pocket is comprised by Helix-14, Helix-15, and Helix-19, a number of hydrophobic residues contributed for the formation of this pocket, including V247, V250, I254, L255, L264, A268, V273, V277, F280, L284, L287, and L316 (Figs. 1D and 4C). However, this hydrophobic pocket in occupied by P protein in NiV NP, but is accommodated by Helix-20, which is consisted by residues from N325 to E351, in EBOV NP_core_. Five hydrophobic residues, i.e. V336, Y340, L343, A346, and A347, stabilize the interaction between Helix-20 with the hydrophobic pocket in C-lobe.

We next investigated the conservation of this hydrophobic pocket among *filoviridae* family. Five strains of Ebola virus (strain *Zaire*, UniProt O72142; *Sudan*, UniProt Q5XX08; *Bundibugyo*, UniProt B8XCM7; *Tai forest*, UniProt B8XCN6; and *Reston*, UniProt Q8JPY1), five strains of Marburg virus (strain *Angola*/*2005*, UniProt Q1PD53; *Ozolin*/*1975*, UniProt Q6UY69; *Popp*/*1967*, UniProt P35263; *Ravn*/*1987*, UniProt Q1PDD0; and *Musoke*/*1980*, UniProt P27588) and one strain of Lloviu cuevavirus (strain *Asturias-Bat86*/*2003*, UniProt G8EFI1) are aligned (Fig. 4D). All of the hydrophobic residues consisting of the pocket are highly-conservative among all the given strains, suggesting the conserved structural architecture of RNP formation among different genus of this family and the critical importance of the function of this hydrophobic pocket.

## DISCUSSION

Structural studies in the past ten years have led an understanding of −ssRNA virus-encoded NPs (Sun et al., 2012; Zhou et al., 2013). Although these NPs possess large variations, their structures can be divided into two topological groups. The first group includes most −ssRNA viral NPs, including BDV from the *Bornaviridae* family (Rudolph et al., 2003), VSV and rabies virus from the *Phabdoviridae* family (Albertini et al., 2006; Green et al., 2006), RSV from the *Paramyxoviridae* family (Tawar et al., 2009), influenza virus from the *Orthomyxoviridae* family (Ye et al., 2006), Rift Valley fever virus (RVFV) (*Phlebovirus* genus) (Raymond et al., 2010), and Bunyamwera virus (BUNV) (*Orthobunyavirus* genus) (Li et al., 2013) within the *Bunyaviridae* family. Although their detailed structures are different, NPs in class I possess a general N- and C-lobe that face each other to form a positively charged crevice for RNA binding but use diverse structural components for the inter-protomer interaction (Sun et al., 2012). NPs in class II include LAFV (*Arenaviridae* family) (Hastie et al., 2011a, b; Qi et al., 2011) and CCHFV (*Nairovirus* genus, *Bunyaviridae* family) (Guo et al., 2012). Although LAFV and CCHFV belong to different virus families and their NPs were found to have additional biological functions, the structural regions for genome encapsidation are highly similar. According to the structural topology, the structure of EBOV NP_core_ belongs to class I viral NP. However, the extension from the core domain to the end of EBOV NP, including a non-conservative region and the C-terminal minor domain which is of about two hundred amino-acid length, distinguishes EBOV NP with other class I viral NPs. Actually, the primary sequence of *Filoviridae* shares high similarity with *Paramyxoviridae* family in the first 450 amino acids, but shows its own specificity for the C-terminal region. The precise biological function of this extension part warrant further investigations.

**Figure 5:**
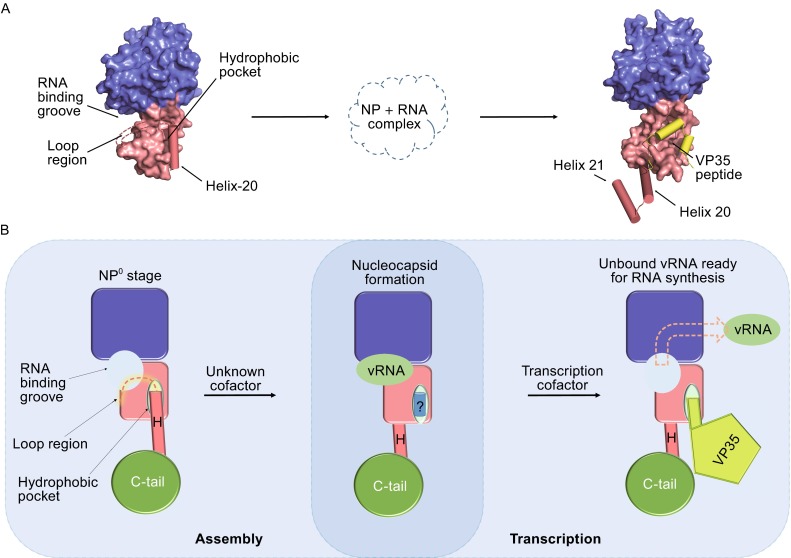
**Structural mobility of EBOV NP**_**core**_ **within virus proliferation**. (A) Structural comparison of EBOV NP_core_ and complex with VP35 peptide. The molecule of EBOV NP_core_ alone and in complex with VP35 peptide are shown in the left and right panels. The N-lobe and C-lobe are colored as blue and red, respectively. Helices 20–21 are shown as cylinder, and the loop region (A326–V334) are linked by dotted lines. Small peptide derived from VP35 is shown as yellow cylinders. (B) A proposed model of conformation change during nucleocapsid assembly and transcription process. RNA binding groove and hydrophobic pocket are indicated by arrow

Although NP is known to be the most, at least one of the most, stable proteins encoded by −ssRNA virus during their proliferation cycle, significant structural shift can be observed in various stages among multiple virus species (Zhou et al., 2013). The most essential results were acquired from the structures of phlebovirus-encoded NPs. The first RVFV NP purified through a denature/refolding method in monomeric form shows a novel compact structure that lacks a positively charged crevice for RNA binding and has no protruding portions for NP oligomerization (Raymond et al., 2010). However, Ferron et al. used a different purification method in physiologically condition to solve the hexameric structure of RVFV NP, which has a highly positively charged inner perimeter as the RNA-binding site (Ferron et al., 2011). Although the body regions of the two structures obtained through different purification methods are identical, significant conformational difference occurs at the N-terminal arm (N-arm). The distinct positions of the N-arm reflect the structural flexibility during RNP formation, in which the monomeric structure may represent a ‘‘waiting’’ conformation before oligomerization and binding with RNA (Ferron et al., 2011). Subsequently, Raymond et al. reported the structures of the NP-RNA complexes of the RVFV and Toscana viruses, other members of the *Phlebovirus* genus, in tetrameric, pentameric, and hexameric forms (Fig. 3D) (Raymond et al., 2012). These structures confirmed that the highly flexible N-arm mediates the contacts between NP protomers, which are responsible for RNP formation (Raymond et al., 2012). This monomeric building block and the flexibility of the NP-NP interaction in the oligomer formation allow RVFV RNP to pack into viral particles with higher structures and density (Raymond et al., 2012). All these results revealed that the component of viral NP could have structural shift in the different stage of virus life cycle to facilitate either RNA binding or further oligomerization to form RNP. During the preparation of this manuscript, Daisy W. Leung et al. reported an EBOV NP structure complex with a peptide from VP35 at 3.7-Å resolution. These two EBOV NP structures may present another essential instance for conformation change during virus life cycle (Fig. 5). In the structure of EBOV NP_core_ alone reported in this work, Helix-20 folds towards and interacts with the hydrophobic pocket on the interface of the C-lobe of EBOV NP_core_ to form a compact structure. In contrast, in the structure of EBOV NP_core_ in complex with VP35 peptide, Helix-20, together with Helix-21, transits to the opposite side of the C-lobe of EBOV NP_core_ (Leung et al., 2015) (Fig. 5B). Because EBOV VP35 peptide (NPBP, residues 20–48) binds NP with high affinity and specificity, inhibits NP oligomerization, and releases RNA from NP-RNA complexes *in vitro*, this structure is likely to represent a transition state immediately before the initiation of viral RNA synthesis in RdRp. We would like to propose that EBOV NP_core_ structure reported in our work is the original state of EBOV NP after it can be translated from host ribosome, which keeps in N^0^ stage, ready for the binding with nascent RNA and the formation of RNP.

Actually, the transition state is very similar to the function of a phosphoprotein (P) in VSV replication (Green and Luo, 2009). P protein is an essential co-factor of VSV RNP. In the structure of VSV RNP-like particle (NP-RNA complex) in complex with C-terminal domain of P, P binds primarily to the C-terminal lobe of two adjacent N proteins within nucleocapsid, and the proximity to RNA cavity indicates that the P protein binding impacts the RNA encapsidation of VSV NP and orients the L protein to access the RNA template without NP (Green and Luo, 2009). Although both P of VSV and VP35-NPBP of EBOV can regulate RNA encapsidation by NPs, significant variations still distinguish them. First, VSV NP can still bind with RNA, even though P protein binds to VSV RNP-like particle, indicating suggesting that the interaction of P with NP cannot directly induce the releasing of RNA in VSV. However, EBOV VP35-NPBP can directly and competitively inhibit the binding of RNA to EBOV NP. Moreover, there is no obvious structural shift of NP component in VSV during P protein binding, but Helix-20 in EBOV NP has significant remodeling with VP35-NPBP interaction. All these differences suggest the potential existence of several other architecture(s) from the original state of EBOV NP to the transition state before RNA synthesis. This warrants further structural and biological validations, such as, the complex structure of EBOV nucleoprotein bound with RNA.

Because the correct RNP formation and function is a key step for the replication, transcription, and assembly for −ssRNA viruses, it is conceivable that blockage of this process would provide great potential for antiviral development. In the last few years, great progress has been achieved based on this new antiviral strategy, particularly for the influenza virus. Through a chemical genetic method, Kao et al. first identified that influenza virus NP is a druggable target (Kao et al., 2010). They reported that nucleozin, a small molecule compound that triggers aggregation and inhibits the nuclear accumulation of NP, can inhibit the replication of influenza virus at a nanomolar median effective concentration (EC_50_) (Kao et al., 2010). In a parallel effort, Gerritz et al. discovered a series of influenza replication inhibitors and showed that they interfere with NP-dependent processes via the formation of higher-order NP oligomers with an EC_50_ up to 60 nmol/L (Gerritz et al., 2011). Notably, the structure of NP in complex with a representative compound of these inhibitors revealed that two inhibitors in an antiparallel orientation lock two adjacent NP protomers. This unexpected quaternary complex explained viral inhibition via the ligand-induced formation of stable NP oligomers (Gerritz et al., 2011). These results cumulatively demonstrated that targeting the formation of viral RNP is a valid goal for the development of small-molecule therapies against viral resistance to currently available drugs targeting surface protein. Here we report a distinct hydrophobic pocket in this work, which shows highly conservative among all viruses within *Filoviridae*. Therefore, the structure of EBOV NP not only aids in understanding the structural and functional differences among NPs encoded by −ssRNA viruses, but also benefits the development of antiviral therapies against EBOV infection.

## MATERIALS AND METHODS

### Protein production

The gene of the *Zaire ebolavirus* nucleoprotein (residues 36–351) was cloned into the pET-21d expression vector within *Nco*I and *Xho*I site following a general protocol, The sequences of the primers are: forward, 5′-CCCATGGCTGTTCGGCAAAGAGTCATC-3′, reverse, 5′-CCCTCGAGCTCAGCCTCAGTGGCAGCCTC-3′. The accuracy of the inserts was verified by sequencing.

The recombinant plasmid of EBOV NP_core_ was transformed into *E. coli* strain BL21 (DE3) and overexpressed as a 6× His tag fused at the C terminus fusion protein. The cells were cultured at 37°C in 800 mL LB media containing 100 μg/mL ampicillin. Once OD_600_ reached 0.6, the culture was transferred to 16°C, and protein was induced by incubating with 0.25 mmol/L isopropyl-β-D-1-thiogalactopyranoside (IPTG) for an additional 18 h. Harvested cells were resuspended in lysis buffer (20 mmol/L Tris-HCl, 500 mmol/L NaCl, pH 8.5) and homogenized with a low-temperature ultra-high pressure cell disrupter (JNBIO, China). The lysate was centrifuged at 25,000 ×*g* for 30 min at 4°C to remove cell debris. The supernatant was then loaded twice onto a Ni-NTA column pre-equilibrated with lysis buffer. Resin was washed four times with 60 mL of wash buffer (20 mmol/L Tris-HCl, 500 mmol/L NaCl, 25 mmol/L imidazole, pH 8.5) and eluted with 30 mL of wash buffer supplemented with 1 mol/L imidazole. The protein was further purified on a Superdex-200 (GE Healthcare) column equilibrated with the buffer containing 20 mmol/L Tris-HCl, 200 mmol/L NaCl, 5 mmol/L DTT, pH 8.5. SDS-PAGE analysis revealed over 95% purity of the final purified recombinant protein. Fractions from the single major peak were pooled and concentrated to 6 mg/mL for crystallization. The purified EBOV NP_core_ was >95% pure according to SDS-PAGE analysis and had an A_280_/A_260_ ratio of 1.6. The purified protein was concentrated to 10 mg/mL and stored at 193 K.

Selenomethionine (SeMet) derivative of EBOV NP_core_ was produced in the methionine-auxotrophic *E. coli* strain B834 (DE3) that was grown in minimal medium supplemented with 3% glucose, 30 mg/L L-selenomethionine, and 100 μg/mL ampicillin. When OD_600_ reached 0.6, the culture was transferred to 16°C, another 30 mg/L L-selenomethionine was added in. Protein was induced by incubating with 0.25 mmol/L IPTG for an additional 24 h. SeMet substituted EBOV NP_core_ was purified following the same condition as wild type protein.

### Crystallization

Initial crystallization trials were performed in a 96-well format using a 1:1 ratio of well solution to protein at 5.5 mg/mL by screening commercial crystal screening kits at 16°C, including the Index, Crystal Screen, PEG/Ion, Salt/RX, Natrix, Crystal Screen Lite, and Crystal Screen cryo from Hampton Research. Small crystals of EBOV NP_core_ first appeared after one day in 200 mmol/L ammonium citrate tribasic pH 7.0 and 20% (*w*/*v*) PEG3350.

Further optimization with additive and detergent screens (Hampton Research) was performed, the final optimized crystal condition was 200 mmol/L ammonium citrate tribasic pH 7.5 and 18% (*w*/*v*) PEG3350. Cubic-like crystals grew to a final size of 40 μm × 40 μm × 60 μm within three days at 16°C. Crystals were harvested and protected in the well solution containing 30% (*w*/*v*) PEG3350 and cooled in dry nitrogen stream at 100 K for X-ray data collection. SeMet EBOV NP_core_ crystals were grown in the same condition.

### X-ray data collection, processing, and structure determination

All crystals were gradually transferred into a harvesting solution containing the respective precipitant solutions plus 5% (*v*/*v*) glycerol before being flash frozen in liquid nitrogen for storage. Data were collected under cryogenic conditions at 100 K. The selenomethionine SAD data set of the EBOV NP_core_ was collected at 2.4 Å using a wavelength corresponding to the Se peak at the SSRF (Shanghai, China) beamline BL19U, and another native data set was collected at 1.8 Å. All data sets were processed using the HKL-3000 package (Minor et al., 2006). The crystals belonged to the space group *P2*_*1*_*2*_*1*_*2* with cell parameters *a* = 59.8 Å, *b* = 162.9 Å, *c* = 31.3 Å, and *α* = *β* = *γ* = 90°. Excluding the first selenium of each polypeptide, 6 of 7 selenium atoms in the asymmetric unit were located and refined, and the SAD data phases were calculated and substantially improved by solvent flattening using the PHENIX program (Adams et al., 2002). A model was manually built into the modified experimental electron density using COOT (Emsley and Cowtan, 2004) and further refined in PHENIX. Model geometry was verified using the program MolProbity (Lovell et al., 2003). The final refinement statistics are summarized in Table 1. Structural figures were drawn using the program PyMOL (DeLano, 2002).

### Accession code

The coordinates and structure factors have been deposited with the RCSB under accession codes: 4Z9P. The authors declare no competing financial interest.

## ABBREVIATIONS

−ssRNA: negative-sense single-stranded RNA
BDV: Borna disease virus
BUNV: Bunyamwera virus
cRNA: complementary positive-strand RNA
CCHFV: Crimean-Congo hemorrhagic fever
EBOV: Ebola virus
GP: glycoprotein
LASV: Lassa fever virus
LLOV: Lloviu virus
MARV: Marburg virus
NP: nucleocapsid protein
RdRp: RNA-dependent RNA polymerase
RNP: ribonucleoprotein
RSV: respiratory syncytial virus
RVFV: Rift Valley fever virus
VP: viral protein
VSV: vesicular stomatitis virus
